# Practice of percutaneous needle autopsy; a descriptive study reporting experiences from Uganda

**DOI:** 10.1186/1472-6890-14-44

**Published:** 2014-12-03

**Authors:** Janneke A Cox, Robert L Lukande, Sam Kalungi, Koen Van de Vijver, Eric Van Marck, Ann M Nelson, Asafu Munema, Yukari C Manabe, Robert Colebunders

**Affiliations:** Department of Clinical Sciences, Institute of Tropical Medicine, Nationalestraat 155, 2000 Antwerpen, Belgium; Infectious Diseases Institute, Makerere University College of Health Sciences, Kampala, Uganda; Department of Pathology, College of Health Sciences, Makerere University, Kampala, Uganda; Department of Pathology, Mulago Hospital Complex, Kampala, Uganda; Department of Diagnostic Oncology & Molecular Pathology, Netherlands Cancer Institute - Antoni van Leeuwenhoek Hospital, Amsterdam, The Netherlands; Department of Pathology, University Hospital Antwerp, University of Antwerp, Antwerp, Belgium; Joint Pathology Center, Silver Spring, USA; Division of Infectious Diseases, Department of Medicine, Johns Hopkins University School of Medicine, Baltimore, Maryland; Faculty of Medicine, University of Antwerp, Antwerp, Belgium

**Keywords:** Needle autopsy, Partial autopsy, Minimal invasive autopsy, Needle biopsy, Ultra-sound guided biopsy, Tru-cut biopsy, Post mortem, Uganda, Sub Saharan Africa

## Abstract

**Background:**

Percutaneous needle autopsy can overcome a number of barriers that limit the use of complete autopsies. We performed blind-and ultrasound guided needle autopsies in HIV-infected adults in Uganda. In this study we describe in detail the methods we used, the ability of both procedures to obtain sufficient tissue for further examination and the learning curve of the operators over time.

**Methods:**

If written informed consent was granted from the next of kin, we first performed a blind needle autopsy, puncturing brain, heart, lungs, liver, spleen and kidneys using predefined surface marking points. We then performed an ultrasound guided needle autopsy puncturing heart, liver, spleen and kidneys. The number of attempts, expected success and duration of the procedure were noted. A pathologist read the slides and indicated if the target tissue was present and of sufficient quality for pathological review. We report the predicted and true success rates, compare the yield of blind to ultrasound guided needle biopsies and evaluate the failure rate over time.

**Results:**

Two operators performed 96 blind needle autopsies and 95 ultrasound guided needle autopsies. For blind needle biopsies true success rates varied from 56-99% and predicted success rates from 89-99%. For ultrasound guided needle biopsies true success rates varied from 72-100% and predicted success rates from 84-98%. Ultrasound guidance led to a significantly higher success rate in heart and left kidney. A learning curve was observed over time with decreasing failure rates with increasing experience and a shorter duration of the needle autopsy.

**Conclusion:**

Needle autopsy can successfully obtain tissue for further pathological review in the vast majority of cases, with a decrease in failure rate with increasing experience of the operator. The benefit of ultrasound guidance will depend on the population, the disease and organ of interest and the local circumstances. Our results justify further evaluation of needle autopsies as a method to establish a cause of death.

## Background

Needle autopsy was first described in 1955 when in 24 corpses tissue was obtained by percutaneous biopsies. Its use was motivated by low acceptance rates and limited accessibility of complete autopsy outside the hospital [1]. Since then, needle autopsy has been used in various settings and for various reasons including increased acceptability, simplicity and decreased risk of disease transmission when compared to complete autopsies [2, 3].

However, an important disadvantage of needle autopsy is the lack of direct visual judgment. This may lead to sampling error of both the target organ and the target lesion. Reported success rates to obtain tissue vary widely depending on the organ involved. The largest postmortem needle biopsy study retrospectively evaluated 394 biopsies that were performed between 1948 and 1968 by 32 different pathology residents in the United States. The success rates ranged from 34% for the kidneys to 92% for the liver [2]. Smaller studies in various adult populations have shown successful biopsy rates of 98-100% for the liver, 76-94% for the lungs, 50-100% for the heart and 10-80% for the kidneys [3–6].

The addition of ultrasound guidance could compensate for the loss of visual judgment. For clinical biopsies in living patients ultrasound guidance is well established and used to increase tissue yield and to prevent complications [7–9]. For needle autopsies, the use of ultrasound guidance was reported in one study [10]. However, since postmortem there is no apparent limit to the number of biopsies and complications no longer need to be prevented, its benefit may be less obvious.

We conducted a study in Uganda in which we subsequently performed blind needle autopsy, ultrasound guided needle autopsy and complete autopsy in deceased HIV-infected hospitalized adults. Our results on the concordance in cause of death between needle and complete autopsy have been published elsewhere [11]. Herein, we report in detail the methods we used, the success rates for the individual organs and the observed learning curve over time. Moreover, we compare the success rates per organ of blind and ultrasound guided needle autopsies.

## Methods

### Setting and population

The study was conducted in Mulago hospital, a tertiary teaching hospital in Kampala, Uganda from February until June 2013. Written informed consent for study participation was obtained from the next of kin of HIV-infected adults (>18 years old) that died during hospitalization. Post-partum deaths and deaths after trauma were excluded.

### General information

The needle autopsy took place within 4 hours after consent was granted. Two medical doctors (JAC, RLL), without previous experience in needle biopsies or ultrasound use performed all needle autopsies in the hospital mortuary. Prior to study initiation, an experienced radiologist instructed the two medical doctors on the performance of ultrasound guided needle biopsies. One automated biopsy needle 14G*16cm (Bard**®** Max-Core**®** disposable core biopsy instrument) was used per patient. For each procedure the start and end-time were recorded. The operator assessed nutritional status on gross examination of the corpse (emaciated, thin, well nourished or obese). After completing all study procedures the body was embalmed free of charge and any needle-entry sites leaking bodily fluids were sutured.

The operator was free to perform as many punctures as needed, however a minimum of 3 attempts and a reasonable maximum of approximately 10 attempts was set. The operator noted the number of attempts and the number of expected successful core biopsies per organ.

The core biopsies (brain, heart, liver, spleen, right and left kidney, right and left lung) were collected in 3 separate specimen containers filled with formalin 10%. For the blind biopsies container 1 collected brain, heart, liver and spleen, container 2 left kidney and left lung and container 3 right kidney and right lung. For the ultrasound guided biopsies container 1 collected heart, liver and spleen, container 2 left kidney and container 3 right kidney.

### Autopsy procedures

For the blind needle autopsy, surface marking points and palpation were used to estimate the location of each individual target organ. The following surface marking points for needle entry were used: the brain through the nose and the cribriform plate, the spleen along the mid-clavicular line under the rib arch pointing the needle in dorso-lateral direction or from the 11^th^ intercostal space mid-axillary in case of a sample error (i.e. no tissue in the biopsy needle), the liver along the mid-clavicular line under the rib arch, both lungs at the level of the 3^rd^ intercostal space below the deltoid tubercle or in case of a sample error from the 6-7^th^ intercostal space mid-axillary, the heart 5^th^ intercostal space lateral from the sternum and both kidneys dorsal below the 12^th^ rib just lateral from the spine. The operator was allowed to change the needle position during the procedure if the macroscopic appearance of the core biopsy would imply so.

Ultrasound guided needle biopsies were taken from the heart, liver, spleen and both kidneys using a pocketsize, portable ultrasound scan (Vscan**®** V1.2, GE Healthcare, USA). The ultrasound scan was used for real-time guidance of the needle to the target organ. However, if the ultrasound scan identified any specific lesion(s) in the target-organ, an attempt was made to also puncture this lesion.

### Histological assessment

For the needle autopsy, one paraffin-embedded tissue block was made per specimen container. Therefore, 6 tissue blocks per patient were made with 4/6 tissue blocks containing core biopsies of more than one organ. An effort was made by the lab-technicians to embed all tissue-cores on the same level, so that when cutting the block all tissue-cores would be present.

From each block, a hematoxylin and eosin (H&E) stained slide was made. A pathologist read each slide and noted per slide if the target tissue was present and of sufficient quality for histological review. If a slide did not contain all the expected tissues, the block was reviewed and when needed re-embedded to ensure all tissue-cores were at one level on the cut-surface. A new H&E stained slide would be made and reread by the pathologist in the same manner as before.

### Definition of outcomes

A successful biopsy was defined as at least one representative core biopsy of a specific organ on the slide where it should be present. This definition was used because within a patient target tissue could be obtained unintentionally when trying to puncture another organ but missed when intending to puncture that organ, e.g. heart tissue could be present on the slide that should contain left lung but not present on the slide that should contain heart tissue. In that case, the heart biopsy was classified as unsuccessful. We report the true success rate, i.e. the proportion of successful biopsies after histological review and the predicted success rate, i.e. the proportion of successful biopsies predicted by the operators during the procedure.

### Statistical methods

Proportions are reported with 95% confidence intervals (CI) and medians with inter-quartile ranges (IQR). When comparing proportions a McNemar test, a Chi square test or a Fisher’s exact test were performed when appropriate. When evaluating the relation between number of attempts and success rates, logistic regression was used. A p-value <0.05 was considered statistically significant. Data were analyzed using STATA version 11.0 (Stata Corp., College Station, TX, Texas, USA).

### Ethics statement

The study received ethical approval from the Joint Clinical Research Center Research and Ethics Committee (Uganda), the Mulago Internal Review Board (Uganda) and the Institute of Tropical Medicine Institutional Review Board (Belgium). The study received final approval and registration by the Uganda National Council of Science and Technology (HS1300).

## Results

We conducted 96 blind needle autopsies and 95 ultrasound guided autopsies; in one patient the ultrasound-guided biopsy was not performed due to generalized cutaneous lesions with extreme desquamation. After processing, 4 kidney-containing ultrasound guided tissue blocks were missing. Therefore we were able to analyze 768 blind biopsies (8 target organs in 96 patients) and 471 ultrasound-guided biopsies (5 target organs in 95 patients, minus 4).

Fifty-seven percent of patients were female, the median age was 35 years (IQR 29–40), 31% were classified emaciated, 38% thin, 27% well nourished and 4% obese. Nine percent of all tissue blocks were re-embedded after initial pathological review. No leakage of fluid from entry-sites was observed and no suturing was performed.

### Blind needle autopsy

The true success rates for the different organs varied from 56-99% (Table 1). The heart had the lowest true success rate, 56% (95%CI 46-66%), followed by the right and left lung, respectively 71% (95%CI 62-80%) and 66% (95%CI 56-75%) and the right and left kidney, respectively 73% (95%CI 64-82%) and 71% (95%CI 62-80%). When combining the biopsies of the left and right lung and the left and right kidney for each patient, the true success rates for the lung increased to 82% (95% CI 75–90) and for the kidney to 83% (95% CI 76-91%).

**Table 1:** **Success rates per organ for blind and ultra-sound guided needle biopsies**

	Blind				Ultrasound			
	Predicted success rate (%, 95% CI)	True success rate (%, 95% CI)	Median# of attempts (IQR)	True success rate/total# of attempts ^§^(95% CI)	Predicted success rate (%, 95% CI)	True success rate (%, 95% CI)	Median# of attempts (IQR)	True success rate/ total# of attempts ^§^(95% CI)
**Brain**	92 (87–98)	93 (87–98)	3 (3–4)	0.27 (0.26-0.29)	-	-	-	-
**Lung right**	99 (97–100)	71 (62–80)*	5 (4–7)	0.15 (0.12-0.17)	-	-	-	-
**Lung left**	99 (97–100)	66 (56–75)*	5 (4–6)	0.13 (0.11-0.15)	-	-	-	-
**Both lungs**	99 (97–100)	82 (75–90)*	10 (8–12)	0.08 (0.07-0.09)	-	-	-	-
**Heart**	97 (93–100)	56 (46–66)*	4 (3–5)	0.14 (0.11-0.16)	91 (85–97)	72 (62–81)*^/^**	3 (3–5)	0.20 (0.17-0.23)***
**Liver**	99 (97–100)	99 (97–100)	3 (3–4)	0.27 (0.26-0.29)	98 (95–100)	100	3 (3–4)	0.29 (0.28-0.30)
**Spleen**	89 (83–96)	76 (67–85)*	5 (3–7)	0.14 (0.12-0.17)	84 (77–92)	72 (62–81)*	5 (3–6)	0.15 (0.12-0.17)
**Kidney right**	91 (86–97)	73 (64–82)*	5 (4–6)	0.15 (0.13-0.17)	91 (85–97)	83 (75–91)	4 (3–5)	0.20 (0.18-0.23)***
**Kidney left**	90 (84–96)	71 (62–80)*	5 (4–6)	0.16 (0.13-0.18)	91 (85–97)	86 (79–93)**	4 (3–6)	0.20 (0.17-0.22)***
**Both kidneys**	97 (97–100)	83 (76–91)*	10 (8–11)	0.09 (0.08-0.10)	96 (92–100)	91 (86–97)	9 (7–11)	0.11 (0.10-0.11)

CI: Confidence interval; #: Number IQR: Inter quartile range.

^§^: Selection of 80 patients for which the number of attempts were noted for each organ. True success rates were not significantly different in these 80 patients when compared to the whole cohort both for blind and ultrasound guided needle biopsies. The minimum number of attempts was 3, therefore the maximum rate is 0.33.

*: Predicted and true success rate significantly different.

**: True success rate significantly different between blind and ultrasound guided needle biopsy.

***: True success rate/total# of attempts significantly different between blind and ultrasound guided biopsy.

The true success rates were significantly lower than the predicted success rates for all organs, except brain and liver. The predicted success rates varied from 89-99% (Table 1).

Because a variable number of attempts were allowed to obtain a biopsy, we calculated the true success rate per attempt (Table 1). For this analysis we selected 80 patients for whom the number of attempts was noted for all 8 target-organs. The true success rates in these 80 patients were not significantly different than the true success rates of the whole cohort. We found no significant correlation between an increasing number of attempts and true success rate in any organ. For the spleen, an increasing number of attempts lead to a lower true success rate (p = 0.003).

There were 188 (24%) unsuccessful biopsies in 68 (71%) patients (Table 2). Thirty-five (19%) of these were predicted by the operator. This could be because of several reasons: the operator could have failed to pierce the cribriform plate in case of a brain biopsy or the macroscopic appearance of the core biopsy made the operator doubt if he/she had punctured the target organ. In 16 (9%) unsuccessful biopsies, the target tissue was present but of insufficient quality for histological review. Patients classified as “thin” had more unsuccessful biopsies compared to all others (p = 0.01).

**Table 2:** **Unsuccessful biopsies for each organ**

	Blind			Ultrasound		
	Total	Predicted n (%)	QNS n (%)	Total	Predicted n (%)	QNS n (%)
**Brain**	7	6 (86)	-	-	-	-
**Right lung**	28	1 (4)	5 (18)	-	-	-
**Left lung**	33	1 (3)	3 (9)	-	-	-
**Heart**	42	3 (7)	2 (5)	27	8 (30)	2 (7)
**Liver**	1	1 (100)	-	-	-	-
**Spleen**	23	7 (30)	-	27	13 (48)	1 (4)
**Right kidney**	26	8 (31)	4 (15)	16	6 (38)	-
**Left kidney**	28	8 (29)	2 (7)	13	5 (38)	-
**Total**	188	34 (18)	16 (9)	83	32 (39)*	3 (4)

n: Absolute number; QNS = Quality not sufficient.

*Significantly different when compared to the proportion of predicted unsuccessful biopsies in the blind needle autopsy.

### Ultrasound guided needle autopsy

For the ultrasound-guided biopsies the true success rate varied from 72-100% (Table 1). The predicted success rates varied from 84-98% and were significantly higher than the true success rate for the heart and the spleen. When considering the same 80-patient subset mentioned before, we found a significant correlation with an increasing number of attempts leading to lower true success rates for the heart, the spleen and right kidney (respectively p = 0.002, p = 0.02 and p = 0.01). Ultrasound imaging detected no specific lesions within the target organs and only once a large abdominal lesion outside a target organ was seen and successfully punctured.

The use of ultrasound guidance led to a significantly higher true success rate for the heart (72% versus 56%, p = 0.03) and the left kidney (86% versus 71%, p = 0.01) when compared to blind biopsy. The true success rate for kidney tissue irrespective of the side was not significantly different between ultrasound guided and blind needle autopsy (91% versus 83%, p = 0.09). Moreover, the true success rate per number of attempts was significantly higher with ultrasound guidance for the heart (0.2 versus 0.13, p = 0.0001) and the right kidney (0.2 versus 0.15, p = 0.002). Ultrasound guided needle autopsy had fewer unsuccessful biopsies (18% versus 24%, p = 0.01) and more expected unsuccessful biopsies (22% versus 39%, p = 0.01), when taking only into account the 5 organs that were punctured in both procedures.

### Experience over time

**Figure 1:**
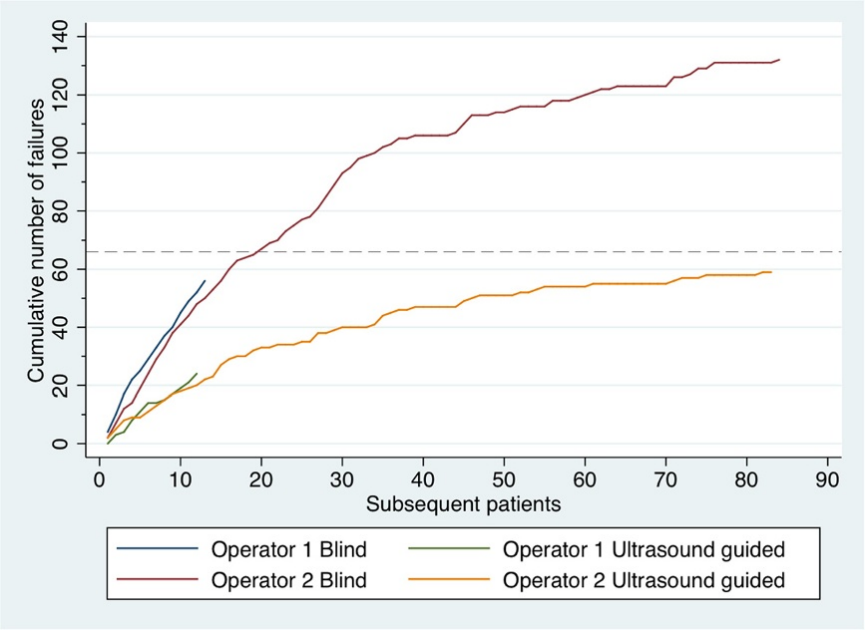
**Cumulative number of unsuccessful biopsies per operator in each subsequent procedure performed.** The dashed line indicates 50% of cumulative unsuccessful biopsies for operator 2 in the blind needle biopsies.

Operator 1 performed 13 (14%) blind and 12 (13%) ultrasound guided biopsies and operator 2 performed the remaining 83 blind and 83 ultrasound-guided biopsies. The mean number of unsuccessful biopsies per patient was similar for both operators when comparing the blind biopsies from operator 1 (4.3, 95% CI 3.6-5.0) to the first 13 blind biopsies from operator 2 (3.8, 95% CI 3.1-4.6) and when comparing the ultrasound guided biopsies from operator 1 (2, 95% CI 1,2-2,8) to the first 12 ultrasound-guided biopsies of operator 2 (1.7, 95% CI 1.1-2.2) (Figure 1). For operator 2, over time the cumulative number of unsuccessful biopsies leveled off; when considering the last 13 blind and last 12 ultrasound guided biopsies, the mean number of unsuccessful biopsies per patient was 0.7 (0.1-1.3) and 0.2 (95% CI 0–0.4) respectively, implying a learning curve over time for both procedures.

For operator 1 and 2, the mean duration of the first 13 blind needle biopsies was 25 min (95% CI 19–31) and 20 minutes (95% CI 18–22) respectively. The last 13 blind needle biopsies took performer 2 on average 16 min (95% CI 15–18). For operator 1 and 2 the mean duration of the first 12 ultrasound-guided biopsies was 24 min (95% CI 18–31) and 23 minutes (95% CI 21–26) respectively. The last 12 blind needle biopsies took performer 2 on average 14 min (95% CI 12–15).

## Discussion

We found postmortem needle biopsies using surface marking-points to be a successful method for obtaining adequate tissue for further pathological review in the majority of cases. Operators tended to overestimate the success of the needle biopsy. The addition of ultrasound guidance lead to higher true success rates and success rates per attempt in the heart and the individual kidneys. A learning curve was observed in the performance of needle autopsy.

The success rates of blind needle biopsies for the different organs are very comparable to the results of others that performed needle autopsy in adults; the heart, the lungs and the kidneys were most difficult to successfully biopsy [2–4, 6]. These organs cannot be easily palpated, which partly explains why they have the lowest biopsy success rates. Any benefit of ultrasound guidance would be expected mainly in those organs and was indeed found for the heart and the individual kidneys. Another study that used ultrasound guidance for postmortem biopsies of spleen (n = 39), kidneys (n = 39) and heart (n = 3) reported a yield of respectively 87%, 100% and 100% [10]. So the tissue yield for certain organs can be increased by the addition of ultrasound guidance. However, ultrasound guidance also has disadvantages; it requires the availability of an ultrasound machine and it decreases the simplicity of the procedure. Moreover, ultrasound guidance is unsuitable for some organs (brain and lungs). Therefore, whether ultrasound guidance should be recommended depends on the setting and the organs of interest. In our population of severely immune-compromised HIV-infected adults the addition of ultrasound guidance did not lead to the identification of more causes of death, however, for other populations e.g. where renal or cardiac diseases are more prevalent, this might be different [11].

The entry points we used rely on normative general anatomy. Alternative entry points have been reported, although their descriptions are vague: a ‘bonesaw tract’ to biopsy the brain and a ‘lateral position’ to biopsy the kidneys [4, 6]. For organs with a relative low yield alternative entry points should be further explored, e.g. below the xyphoid pointing the needle towards the left axilla for the heart or the 9^th^ or 10^th^ intercostal space behind the mid axillary line for the spleen. Moreover, to evaluate and compare needle autopsy it is important to know what exact methods are being used. Detailed methodological descriptions, e.g. what needle entry points are used, how many punctures are performed per organ, how tissue collection and processing takes place, should therefore be reported in needle autopsy studies.

We had set a reasonable maximum of 10 biopsy-attempts per organ and often far fewer attempts were made. A way to increase the yield could be to increase the number of attempts. However, we found for some organs a decrease in success rate with an increasing number of attempts, implying more punctures were done on ‘difficult’ organs without increasing the number of successful punctures. Therefore, merely increasing the number of attempts will unlikely increase the number of successful biopsies. Changing the entry point and/or position during the procedure may increase the success rate. This information was not collected during our study. Moreover, we found that predicted success rates did not correspond well to the true success rates. Therefore, the judgment of the operator should not be used to guide the sampling. A possible way forward to increase tissue yield would be to biopsy organs that are notoriously difficult more often and use different entry point and/or needle positions for each subsequent attempt*.*

Others showed the degree of experience and motivation of the person performing the biopsies influenced the yield [2]. We also observed a clear learning curve over time. Moreover, the procedure was fast, the visible marks after the needle autopsy were limited and no suturing of needle entry points was needed. Although medically trained people performed the needle autopsies, they were laymen in the field of needle biopsies. This offers perspectives for needle autopsies to be performed on a broader scale. Potential possibilities include practice outside a hospital setting by trained lower level healthcare workers.

This study has several limitations. The reported learning curve over time is based on the performance of 1 operator. Moreover, the subsequent performance of the blind, ultrasound guided and complete autopsy may have influenced the learning curve, for example the macroscopic assessment of the organs during the complete autopsy gave the operator feedback on the successfulness of the needle autopsy. Also, the increased failure rate in thin but not in emaciated patients was significant but difficult to interpret. The assessment of the nutritional status might have been inaccurate and also the numbers of patients in the categories “well nourished” and “obese” were small. These results should therefore be interpreted with caution. Finally, we assumed the absence of target tissue was the result of a failed biopsy. However, some small, fragile tissue cores may have also got lost during tissue processing.

## Conclusion

We found that the use of surface marking points led to adequate tissue cores for further pathological review in the vast majority of organs assessed. Whether these will be adequate to diagnose the actual underlying causes of death depends on the population and the diseases and organs involved. However, the ability to obtain adequate tissue biopsies justifies further evaluation of this technique that overcomes many of the barriers of complete autopsy practices.
